# Amino Acids as Building Blocks for Carbonic Anhydrase Inhibitors

**DOI:** 10.3390/metabo8020036

**Published:** 2018-05-24

**Authors:** Niccolò Chiaramonte, Maria Novella Romanelli, Elisabetta Teodori, Claudiu T. Supuran

**Affiliations:** Department of Neuroscience, Psychology, Drug Research and Child’s Health, Section of Pharmaceutical and Nutraceutical Sciences, University of Florence, Via Ugo Schiff 6, 50019 Sesto Fiorentino, Italy; novella.romanelli@unifi.it (M.N.R.); elisabetta.teodori@unifi.it (E.T.); claudiu.supuran@unifi.it (C.T.S.)

**Keywords:** carbonic anhydrase, enzyme inhibition, metalloenzymes, amino acid, glaucoma, tumors

## Abstract

Carbonic anhydrases (CAs) are a superfamily of metalloenzymes widespread in all life, classified into seven genetically different families (α–θ). These enzymes catalyse the reversible hydration of carbonic anhydride (CO_2_), generating bicarbonate (HCO_3_^−^) and protons (H^+^). Fifteen isoforms of human CA (hCA I–XV) have been isolated, their presence being fundamental for the regulation of many physiological processes. In addition, overexpression of some isoforms has been associated with the outbreak or progression of several diseases. For this reason, for a long time CA inhibitors (CAIs) have been used in the control of glaucoma and as diuretics. Furthermore, the search for new potential CAIs for other pharmacological applications is a very active field. Amino acids constitute the smallest fundamental monomers of protein and, due to their useful bivalent chemical properties, are widely used in organic chemistry. Both proteinogenic and non-proteinogenic amino acids have been extensively used to synthesize CAIs. This article provides an overview of the different strategies that have been used to design new CAIs containing amino acids, and how these bivalent molecules influence the properties of the inhibitors.

## 1. Introduction

The interconversion between CO_2_ and HCO_3_^−^ is fundamental for the successful flow of biochemical processes in all living cells [1]. Metabolic conversion is ensured by the hydration of carbonic anhydride which leads to the corresponding soluble ion, bicarbonate, and the release of a proton [2,3]. Due to the high number of physiological process in which these chemical species are essential, the optimal balance between them is vital for all life forms [2,4,5,6,7]. This equilibrium is regulated by a superfamily of metalloenzyme, the carbonic anhydrases (CAs, EC 4.2.1.1) [1]. By accelerating this normally slow reaction [8], CAs fulfil the metabolic needs connected to CO_2_/bicarbonate and protons [2,4,6]. In fact, CAs are among the most effective catalysts known in nature [3] as their turnover number (k_cat_) can reach the value of 10^6^ s^−1^ [9]. Seven genetically different families of these ubiquitous enzymes were identified (α–η) [2]; all of them possess a bivalent metal ion fundamental for catalysis, as the apoenzyme is devoid of activity [3]. Mammalian CAs belong to the α-family and they are characterized by the presence of a bivalent zinc ion within the active site [1]. This Zn^2+^ coordinates a water molecule, or a hydroxide ion in the activated form of the enzyme, making the hydration of CO_2_ a fast process [1,2,10]. 

Human carbonic anhydrases (hCAs) are widespread in the organism, varying for tissue distribution and subcellular localization. Fifteen α-isozymes (hCA I–XV) have been isolated and characterized so far, but only 12 of them are catalytically active [4,5,11]. hCAs are involved in pH and CO_2_ homeostasis but also in the regulation of many crucial physiological processes, such as gluconeogenesis, lipogenesis or electrolyte secretion in a variety of tissues and organs [2,4,5,6]. hCA I and II are the widest expressed isoforms and, together with hCA IV, were identified in the anterior chamber of the eye, being responsible in this organ for the production of bicarbonate, the main constituent of aqueous humor [5,12]. hCA IX and XII can be defined as tumor-associated proteins, due to their massive expression in many hypoxic cancers [4,5,13,14]. These isozymes are transmembrane proteins, with an extracellular catalytic domain; they are fundamental for the survival of tumor cells under stressful conditions, due to their ability to generate a differential pH microenvironment, resulting in increased tumor growth [5,13,15,16]. While CA activators do not have for the moment any approved pharmacological application [17], CA inhibitors (CAIs) are commonly used in therapy, mainly as anti-glaucoma agents, anti-epileptics and diuretics, while other therapeutic applications are still under investigation (anti-cancer, anti-obesity agents, and others) [5,12,18,19,20,21].

## 2. Carbonic Anhydrase Inhibitors (CAIs)

**Figure 1:**
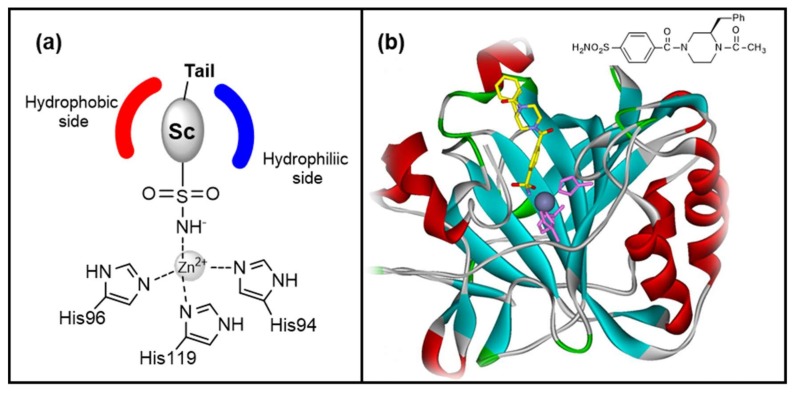
(**a**) Schematization of the interaction between a generic sulfonamide and the Zn^2+^ in the carbonic anhydrase (CA) active site. Sc = Scaffold (**b**) X-ray structure of (*S*)-4-(4-acetyl-3-benzylpiperazine-1-carbonyl)-benzenesulfonamide (**49b**, discussed in Section 7, shown with carbon atoms in yellow,) bound in the active site of hCA I (Protein Data Bank (PDB) entry 6EVR) [23]. The sulfonamide group interacts with the Zn^2+^ (dark grey sphere) which is coordinated by three His residues (in purple).

In the CAs active site the bivalent metal ion normally possesses a tetrahedral geometry, since it interacts with three amino acids moieties and coordinates the water molecule/hydroxide ion. In α-CAs, the Zn^2+^ ligands are three histidine residues (Figure 1) and, together with other key amino acids, they form a very particular and strictly conserved catalytic site within each family of carbonic anhydrase [1,2,3]. It is, therefore, possible to recognize two different regions in the cavity of the active site, a first half composed exclusively of hydrophobic amino acids and a second one where only hydrophilic residues are present [22], leading to an amphiphilic site architecture [2].

**Figure 2:**
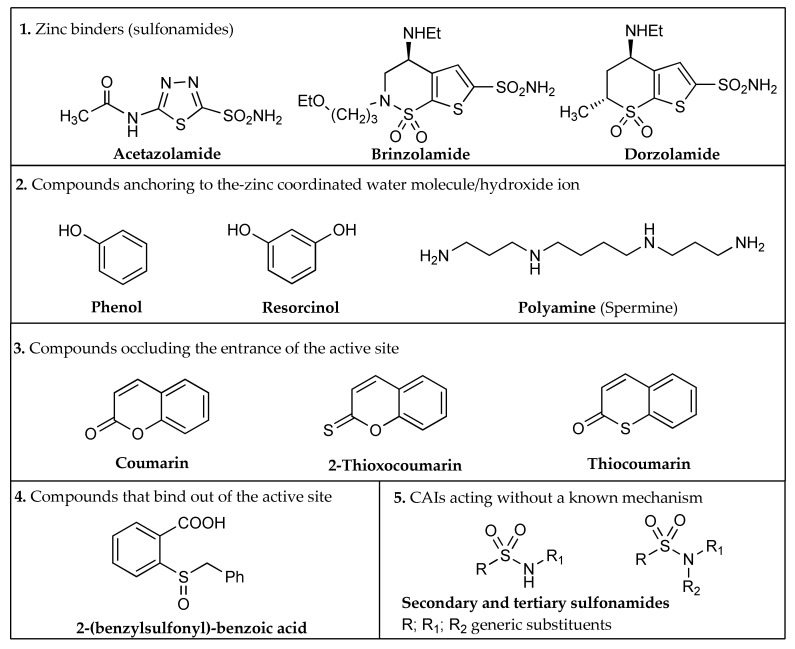
The five classes of carbonic anhydrases inhibitors (CAIs).

Depending on the mechanism of action, five different classes of CAIs are known (Figure 2) [21,24]:

1. Zinc binders, i.e., compounds that chelates the bivalent metal ion of the active site. This interaction interrupts the coordination between the Zn^2+^ atom and the water molecule/hydroxide ion and consequently blocks the enzymatic activity [21,24,25]. The mechanism is schematized in Figure 1a: the scaffold of these molecules (reported as “**Sc**”) may interact with one or both the halves of the active site, stabilizing the interaction with the ion in a tetrahedral geometry. This is the most important class of inhibitors, to which belong sulfonamides and their isosteres (sulfamates or sulfamides), dithiocarbamates, hydroxamate, etc. [3,24]. Sulfonamides are the most widely studied CAIs with at least 20 compounds in clinical use for decades [24]. Some examples (acetazolamide, brinzolamide and dorzolamide) are shown in Figure 2.
2. Compounds that anchor to the-zinc coordinated water molecule/hydroxide ion, such as phenols and polyamines [3,24,26].
3. Compounds occluding the entrance of the active site (coumarins and their isosters) [3,24,27].
4. Compounds that bind out of the active site, such as 2-(benzylsulfonyl)benzoic acid [24,28].
5. CAIs acting without a known mechanism, such as secondary/tertiary sulfonamides, imatinib, etc. [3,24].

As we can note in Figure 2, the five types of inhibitors possess various structures; chemical differences in their pharmacophoric moieties result in different structure activity relationships. Despite this premise, a common problem of CAIs, and in particular of zinc binders, is the lack of selectivity for a specific isoform or the absence of a good water solubility. Many efforts have been made in the last few decades to explore chemical modifications of the CAI structures that could have a positive effect on these properties. In particular, the aim of this review is to provide an overview of the investigated applications of amino acid moieties in the resolution of these problems. This paper is focused on CAIs that carry amino acids or their derivatives in their structures, and how these molecules influence the properties of these inhibitors. Both proteinogenic and non-proteinogenic amino acids have been considered in this analysis and, depending on their synthetic use, five different strategies have been identified.

Amino acids are the primary building blocks of proteins and, of the over 300 naturally occurring, 22 constitute the monomer units of these biological molecules [29]. Amino acids are widely used in organic chemistry; they constitute the smallest fundamental pieces in solid phase peptide synthesis but, due to their characteristics, they are also commonly used in solution synthesis [30]. Their properties range from acidic to basic due to the contemporary presence of a carboxyl and an amino group that, in addition, allow the possibility to easily generate different kind of connections to link the amino acid to other molecules. Beside this, a number of amino acids have reactive groups in their side chains that can be chemically modified [29]. Furthermore, the use of natural α-amino acids allows a broad range of derivatives to be obtained, with the possibility, starting from enantiopure amino acids, to further define the stereochemistry of the side chain. Thanks to ionizing properties that could be exploited to form salts, the carboxy and the amino moieties also ensure good water solubility to these derivatives.

## 3. Amino Acyl as a Water-Solubilizing Tail

Glaucoma is a group of optical neuropathies associated with progressive loss of visual field, leading to visual impairment and blindness. There is a general agreement that increased intraocular pressure (IOP) is the most important risk factor for the outbreak and progression of this disease [12,31]. It was demonstrated [32] that in glaucomatous patients the inhibition of CAs leads to a reduction of IOP and an amelioration of the symptoms. Due to the wide distribution of the different CA isozymes in the body, the administration of systemic inhibitors usually elicits undesired side effects [12,31,33]. For this reason, the treatment of glaucoma is mainly performed with topically administered CAIs [12,34]; two drugs, brinzolamide and dorzolamide (Figure 2), are available in the clinic. These two inhibitors often produce local side effects, such as ocular burning, a stinging sensation, superficial punctuate keratitis, blurred vision and reddening of the eye. These problems are mainly due to the strong acidic pH of their solution, as they are both administered as hydrochloride salts [12,35].

In order to reduce these inconveniences, many efforts have been made to develop new topically administered CAIs. Among others, a method investigated in depth has been the attachment of water-solubilising tails to known effective CAIs. This method is particularly important for sulfonamides, very potent anti-glaucoma agents often endowed with low water-solubility.

**Figure 3:**
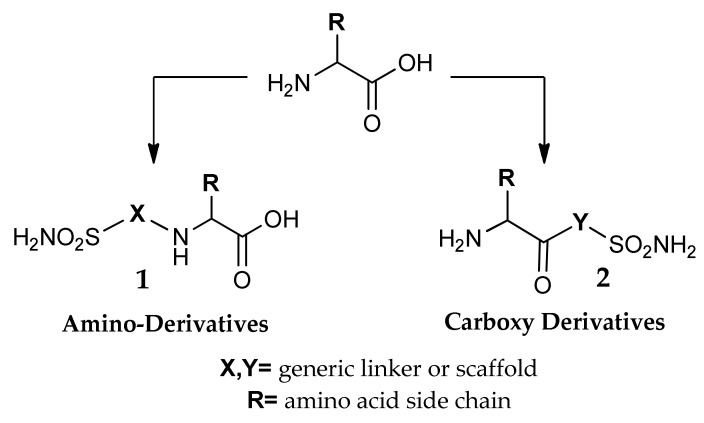
General structure of amino acids functionalised on the amino group (**1**) or on the carboxy moiety (**2**).

The use of amino acids for this purpose was investigated by many research groups, with a broad variety of approaches. The bivalent chemical nature of these synthons allows two possible series of products to be obtained, the amino-substituted compounds **1** and the carboxy derivatives **2** (Figure 3). In **1**, the free carboxyl group can be treated with strong bases to give salts or can be further functionalized. Also compounds **2** can be endowed with good hydrophilic properties thanks to the free basic amino group: such compounds are ideal for ophthalmologic applications, since the water solutions of their salts with strong acids have a weakly acidic pH, preferred over those of alkaline pH [36]. 

**Figure 4:**
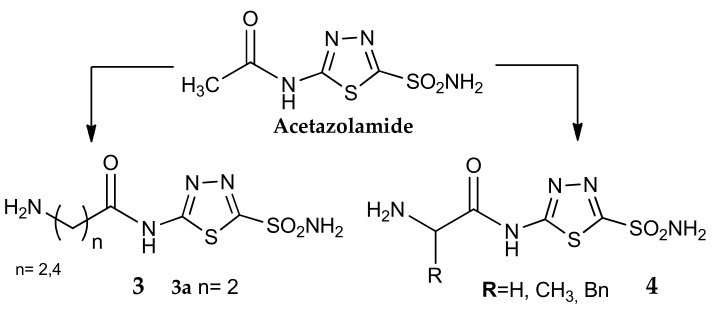
Structure of acetazolamide analogues **3** and **4**.

Antonaroli et al. [37], Blackburn’s [38] group, and later Barboiu et al. [39] reported some structural manipulations of acetazolamide, where the N-acetyl residue was substituted with different aminoacids, giving compounds with general formula **3** and **4** (Figure 4). The structural modification of this first-generation CA inhibitor led to interesting products: the salts of compounds **3** and **4** showed a very good water solubility, associated with effective inhibitory activities against the enzyme. As an example, the β-alanyl derivatives **3a** was three times more potent on hCA II and over 100 times on hCA I than dorzolamide. Only against the isozymes hCA IV, this compound showed an activity three times lower than the reference drug.

**Figure 5:**
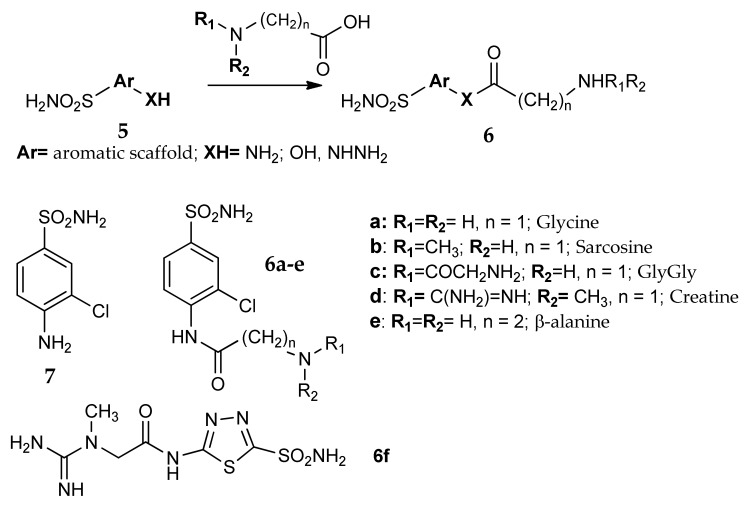
General structure and some examples of the compounds investigated by Scozzafava et al. [36,40].

A similar approach was also investigated by Scozzafava et al. in two separate works [36,40]. Twenty six different sulfonamides containing amino, imino, hydrazino or hydroxyl groups (general formula **5**, Figure 5) reacted with the carboxy moiety of five different glycine derivatives (glycine, sarcosine, creatine, gly-gly and β-alanine). The extremely versatile nature of the carboxyl group allowed a large library of new compounds (general formula **6**) to be generated, differing not only in the structure of the two reagents, but also for the characteristics of the new formed carboxy-derived bonds (i.e., amidic, esteric and hydrazidic bonds).

All the 130 obtained compounds **6** possess very good water solubility as salts of strong acids. They showed a wide range of inhibitory activities against three CA isozymes, hCA I, II and bCA IV. The addition of the aminoacyl/dipeptidyl moiety generally led to an increase of CA inhibitory properties with respect to the corresponding parent sulfonamide. The five amino acid derivatives **6a**–**f** can be taken as an example: they were from 3 to 18 times more potent than the precursor **7**. In addition, the most active compounds of the series were selected for in vivo studies. The topical application directly into the eye showed IOP-lowering effects both in normotensive and glaucomatous rabbits, a frequently used animal model of glaucoma [41,42]. As an example, the acetazolamide derivative **6f** was a more efficient and long-lasting IOP-lowering agent compared to the reference drug dorzolamide.

**Figure 6:**
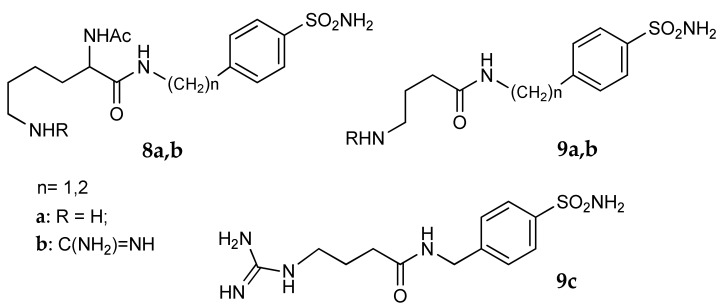
Structure of benzenesulfonamide derivatives **8** and **9**.

It is also possible to enhance the basic nature of the free NH_2_ group by conversion into a guanidine moiety, as reported by Ceruso et al. [43]. The carboxyl group of two amino acids, Nα-acetylysine and γ-aminobutyric acid, was connected to a benzyl or phenetylamine carrying a sulfonamide group, obtaining a first series of basic compounds (**8a** and **9a**, Figure 6); subsequently the amino groups were treated with *N*,*N*′-di-Boc-*N*″-trifluoromethane-sulfonylguanidine, obtaining the guanidine derivatives **8b** and **9b**. This transformation enhanced the solubility of the hydrochloride salts but not their potency, since the guanidine derivatives showed Ki values almost in the same range of the amino-precursor on hCA I, hCA II and *Porphyromonas gingivalis* γ-CA. The same products were also tested against the two tumor-associated isoforms hCA IX and hCA XII [44]. Due to their transmembrane localization and their extracellular catalytic domain, the use of hydrophilic CAIs, endowed with poor cellular permeability, could in principle selectively target these isoforms. The effect of the terminal guanidine moiety on activity was relevant only on hCA XII for some GABA-derivatives: as an example, **9c** showed a K_i_ value almost nine times lower than the corresponding amino precursor.

**Figure 7:**
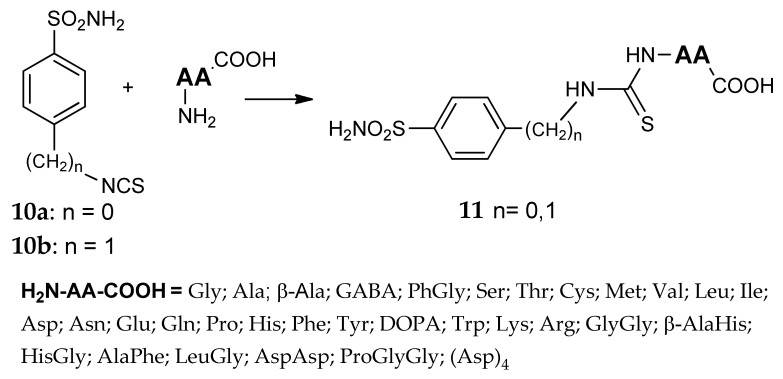
Thiourea derivatives studied by Casini et al. [35,45].

The addition of the amino acid by means of the amino group, instead of the carboxy one, was investigated by Casini et al. in two separate, but closely related papers [35,45]. Two series of sulfonamides were synthesized by the reaction of 4-isothiocyanatobenzenesulfonamide **10a** or 4-isothiocyanatomethyl-benzenesulfonamide **10b** with 33 different amino acids and oligopeptides, forming a thioureido linkage between these two portions (general formula **11**, Figure 7). Many newly obtained compounds possess very good water-solubility properties; the presence of at least one free carboxyl group allows the easy formation of sodium salts. Their solutions showed pH values in the range of 6.5–7.0 and, due to these optimal values, no or modest eye irritation effects were observed. Strong inhibitory properties were detected for many derivatives against hCA I, hCA II and bCA IV and, thanks to their strong tendency to concentrate in ocular fluids and tissues, almost 20 compounds showed effective IOP-lowering properties after topical administration.

**Figure 8:**
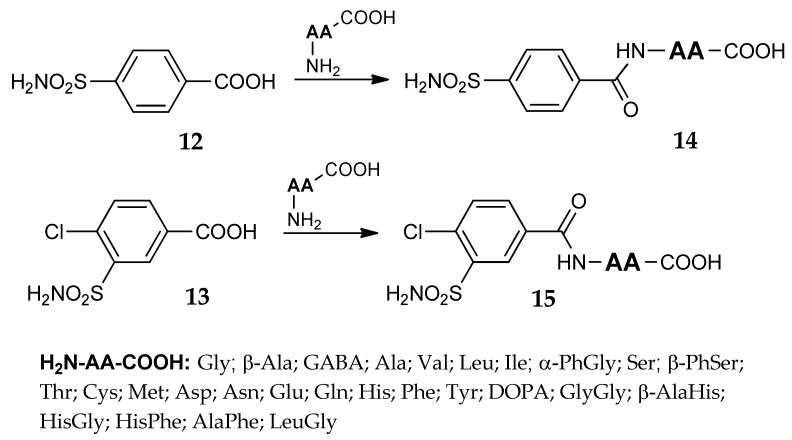
Sulfamoylbenzamides of amino acids and dipeptides.

Using 4-carboxybenzenesulfonamide **12** or 4-chloro-3-sulfamoylbenzoic acid **13** (Figure 8) Mincione et al. [46] synthesized a new series of inhibitors, where different amino acids or dipeptides were directly linked to the sulfonamide through an amidic bond (general formula **14** and **15**, Figure 8). As previously mentioned for the thioureido derivatives **11**, many newly obtained compounds were able to inhibit three CA isozymes (hCA I, hCA II and bCA IV) showing K_1_ values in the nanomolar range. The corresponding carboxylate salts, with their good water-solubility, were investigated in IOP-lowering in vivo experiments. The topical administration of these solutions, which possessed pH values in the neutrality range, showed very important and long lasting IOP-lowering effects in rabbits, stronger and longer-lasting on the glaucomatous animals than on the normotensive ones. 

## 4. The Tail Approach to Carbonic Anhydrases Inhibitors Using Amino Acids

**Figure 9:**
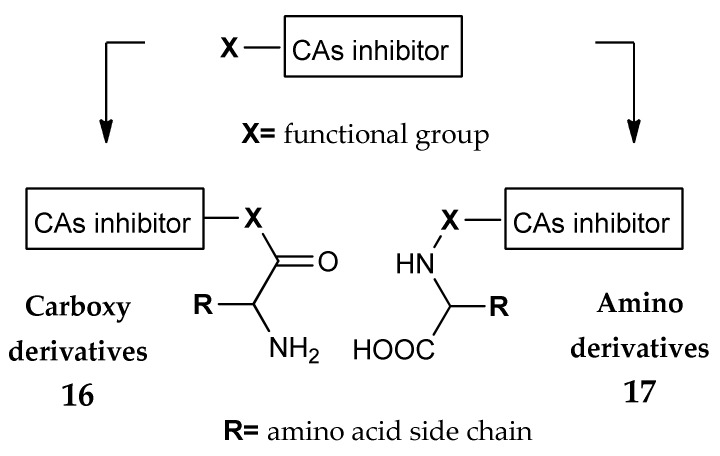
General structure of CAIs decorated with amino acids through the carboxy group (**16**) or the amino moiety (**17**).

As reported in section 2, CAIs fall into five categories, according to their mechanism of action [21]. Probably, the most important CAIs problem is the lack of selectivity for a specific isoform, due to the well conserved catalytic site among the isozymes. A possible method to gain selectivity is the so called “Tail Approach” [21,24,25,33]. This strategy is based on the structural modification of a portion on the inhibitor (Tail in Figure 1a), usually in a position not fundamental for the Zn-chelating activity, that could potentially interact with the regions surrounding the active site, where the amino acids variability is higher. On this basis, the chemical differences in the amino acids’ side chains and the possibility to use also “non-α” amino acids make these molecules optimal candidates for the “tail” role (Figure 9). As is simple to imagine, there is an extremely high variety of possible synthetic pathways that can be used to obtain different compounds by using the carboxy (as in **16**) or the amino moiety (as in **17**).

**Figure 10:**
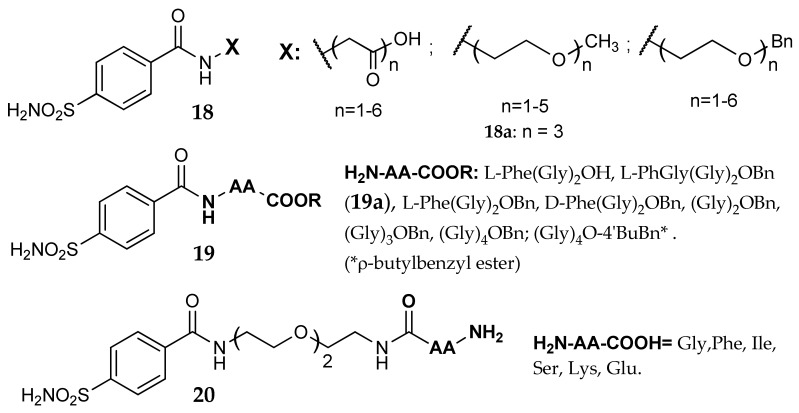
General structures of the compounds investigated in [47,48,49].

An interesting work, focused on the investigation of enzyme-inhibitor interactions, was performed by the groups of Whitesides and Christianson [47,48,49]: in order to explore if secondary interactions away from the active site could influence inhibitory activity, they prepared three series of derivatives (general formula **18**–**20**, Figure 10). At first these researchers synthesized a series of oligolgycine- and oligo(ethy1eneglycol)-linked benzenesulfonamides (**18**), whose dissociation constant on bCA II (K_d_, measured by means of a competitive fluorescence-based assay) was found in the high micromolar range and independent of the polymer length [48]. Later, they condensed several tripeptides to 4-sulfamoylbenzoic acid, obtaining compounds with general formula **19**, endowed with K_d_ values in the nanomolar range on hCA II [49]. The complex with **19a** was analyzed by means of X-ray crystallography: the phenylglycine moiety was placed near Phe131 and Pro202, establishing hydrophobic interactions and validating the initial design. 

The compound carrying three ethylene glycol moieties (**18a**) was, therefore, chosen by Boriack et al. as primary scaffold for their final investigation [47]. Different amino acids were added to the terminal portion of the poly-ethylene glycol chain; with this design compounds **20** were obtained, carrying a free amino group, and endowed with increased activity (K_d_ values from 2 to 10 times lower than the parent compound **18a**). This flexible and quite long linker was able to direct the amino acid toward enzyme surfaces far from the zinc ion, in a region where it can stabilize the binding through side interactions. Since in this series of compounds the best terminal pendants were lipophilic amino acids, these researchers suggested that the linker chain delivered the terminal group close to a hydrophobic region composed by Pro201, Pro202 and Leu198, even if the X-ray structures (Protein Data Bank (PDB) codes 1CNW, 1CNX and 1CNY) did not reveal ordered folds of the ethylene glycol linker. 

**Figure 11:**
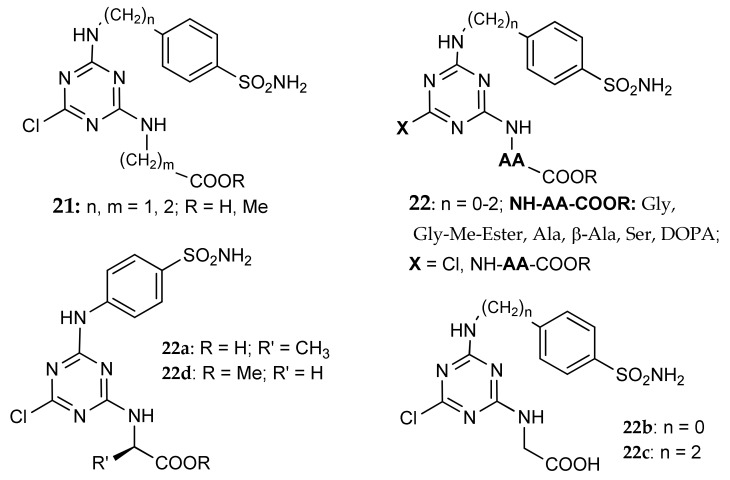
General structure of triazinyl derivatives **21**-**22.**

Garaj et al. [50] used the tail approach on a series of novel sulfonamides incorporating the 1,3,5-triazine moiety, and obtained a small set of compounds with affinity from nanomolar to micromolar on hCA I, hCA II and hCa IX. In this series, glycine (m = 1) and β-alanine (m = 2) derivatives **21** (Figure 10) showed good potency on hCA IX, with Ki values 20–30 times lower than on hCA I and II, but independently of m. The library of these derivatives was later expanded by Carta et al. [51], who investigated, among several substituents, a wider number of amino acids in position 2 or 2,4 of the triazinyl ring (general formula **22**, Figure 11). Many compounds showed nanomolar K_i_ values against the transmembrane tumor-associated CA IX and XII, in addition to CA XIV; the latter isoform is not associated with cancers but is widespread in many tissues such as the kidney, liver and brain among others [3]. As for **21**, higher K_i_ values were found against the off-target isoforms hCA I and II. In some cases some selectivity was obtained: as an example, compound **22a** showed a Ki 0.96 nM on hCA IX, being on this isoform 6, 10, >550 and >1300 times more potent than on hCA XII, XIV, II and I, respectively. Two derivatives (**22b** and **22c**) displayed significant activity also on hCA VII. The binding of the analogue **22d** was analyzed by means of X-ray crystallography (PDB 2ILI) on hCA II: the chlorine atom was engaged in contacts with the side-chain atoms of Ile91 and Gln92 while the triazinyl ring made π-stacking interactions with the phenyl ring of Phe131. The glycine moiety was directed toward the rim of the active site, apparently not engaged in positive interactions. 

**Figure 12:**
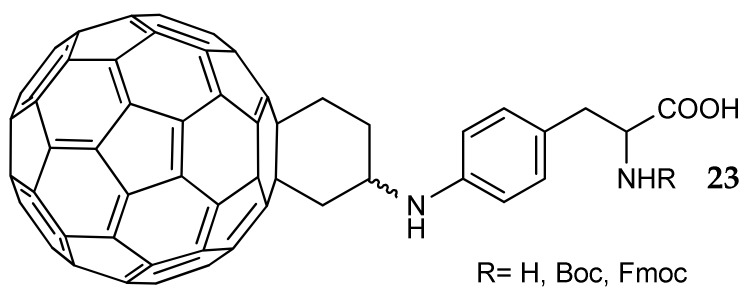
Fullerene-amino acid derivatives studied in [52].

Amino acids were the ideal candidates for the side decoration of fullerene (C_60_), an atypical potential CA inhibitor [52]. Fullerene derivatives possess some interesting properties: as an example, fullevir [53], the sodium salt of fullerene-polyhydro-polyaminocaproic acid, displays significant antiviral, antibacterial and anticancer activity [54,55,56,57]. In addition, fullerene possesses a diameter of about 1 nm, a size similar to the width of the active site entrance of most CA isozymes [58]. In order to improve the very low water solubility and to insert groups that could provide selectivity by interacting with residues of the entrance of the catalytic site, some phenylalanine derivatives were investigated as fullerene pendants (general formula **23**, Figure 12). Inhibitory activity was measured on a panel of hCA isozymes (I, II, III, IV, VA, VB, VI, VII, IX, XII, XIII, XIV, XV), finding K_i_ values in the micromolar range and no selectivity. Computational studies suggested that the inhibition mechanism is the occlusion of the active site through the fullerene cage; it is, therefore, possible that this bulky and extremely rigid scaffold prevents pendants from interacting with crucial amino acid residues, located in a deeper area of the enzyme. This hypothesis seems confirmed by the very low difference in activity measured for the synthesized fullerenes, including those not carrying amino acids pendants.

**Figure 13:**
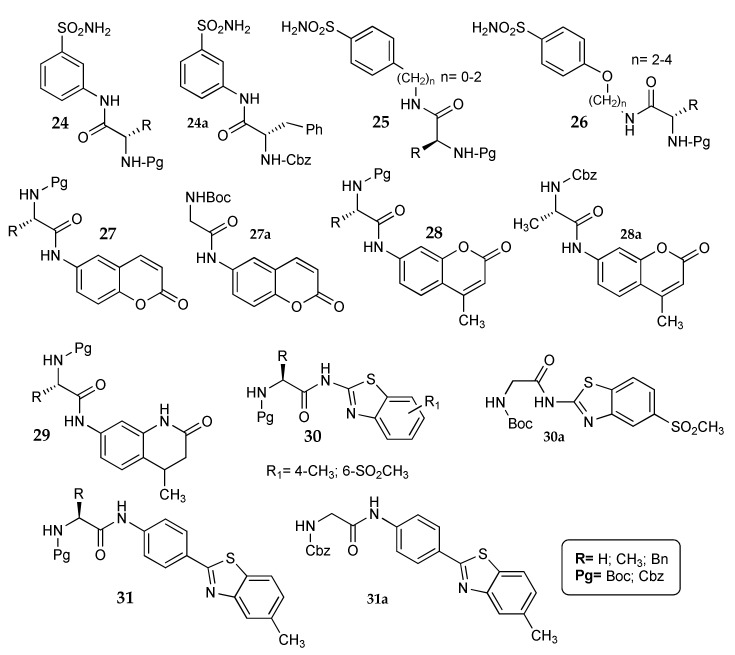
Structure of the compounds investigated by Küçükbay et al. [59,60,61].

A wide investigation of the “amino acid-tail approach” was performed by Küçükbay et al. They synthesised carboxy-derivatives linking a N-protected amino acid to three different types of CA inhibitors, obtaining amino-sulfonamides (**24**–**26**) [59], coumarins (**27**, **28**), tetrahydroquinolinones **29** [60] and benzothiazoles **30**, **31** [61] (Figure 13).

Compounds **24**–**31** were tested against four CA isozymes (hCA I, II, IV and XII). The results were extremely different between the three series. Sulfonamides **24**–**26** were the most active and sensitive to structural variation, with K_i_ values mainly in the nanomolar range. By analysing the data, it is indeed possible to note appreciable differences in activity when the N-protected amino acid was varied, even if it is not simple to infer clear structure activity relationships. Few compounds were endowed with some selectivity toward CA XII: on this isoform, for instance, compound **24a** (K_i_ 9.5 nM) is 18, 47 and 201 times more potent than on CA IV, II and I, respectively.

Dihydroquinolinones **29** were inactive on the four isoforms, while coumarins **27** and **28** showed micromolar K_i_ values only against hCA IV and XII [60]. As a matter of fact, this scaffold is already known as preferential inhibitor of the tumor-associated hCA IX and XII [24,62,63]; the addition of an amino acid tail abolished activity against the off-target isozymes hCA I and II. Moreover, two derivatives (**27a** and **28a**, Figure 13) were completely inactive also against hCA IV, thus showing selectivity for hCA XII. 

Benzoathiazoles **30** and **31** showed micromolar K_i_ values almost exclusively on the ubiquitous isoform hCA I, with only three compounds being active also on hCA II. Surprisingly, one N-Boc-glycine derivative (**30a**, Figure 13) showed activity also on hCA XII, with a K_i_ value nine time lower than that registered against hCA I. Another compound, **31a**, showed activity only on CA II, albeit with low potency (K_i_ 10 μM). 

It is interesting to note that compounds **24**–**31** inhibit CA by different mechanisms, owing to the diverse scaffolds carrying the amino acid tail. The analysis of this research suggests that the “amino acid tail approach” could be interesting for the derivatization of different kind of CA inhibitors. The pretty simple and quick synthetic methods available to link an amino acid moiety to a generic group allow a large library of structurally-related compounds to be obtained, from which it is easy to study the effects of the substitution on activity. In addition, the hydrophilic nature of these molecules could be useful for enhancing their water-solubility. 

## 5. Amino Acids as Linkers

**Figure 14:**
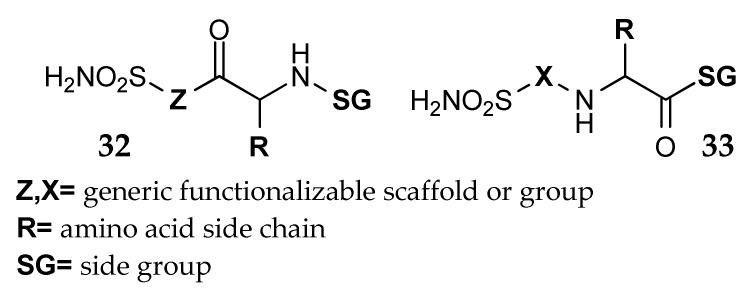
General structure of sulfonamide CAIs having an amino acid as linker.

The bifunctional chemical nature of the amino acids can be used to link together two different substituents, generating molecules with new characteristics. A generic sulfonamide could indeed be connected to another chemical entity through these bivalent linkers (generic formula **32** and **33**, Figure 14). The possibility to protect the amino or the carboxyl moieties, in addition to their different reactivity, allows the easy and selective functionalization of these chemical groups. This strategy could be considered an upgrade of the “amino acid tail approach”, on these compounds, beyond the amino acid chain, the side group (**SG** in Figure 14) represents an additional point of variation.

**Figure 15:**
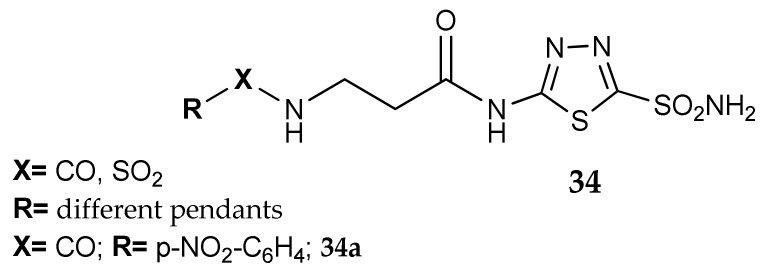
Acetazolamide derivatives studied by Barboiu et al.

A first example is the previously cited work of Barboiu et al. [39], where compound **3a** (Figure 4) was further functionalized on the free NH_2_ with various pendants, affording a wider series of 5-substituted thiadiazole-2-sulfonamides (general formula **34**, Figure 15). In order to obtain derivatives with different physico-chemical properties, compound **3a** (Figure 4) was reacted with 30 different electrophilic reagents (i.e., alkyl/aryl sulfonyl chlorides or fluorides, sulfonic acid cyclic anhydrides or acyl chlorides) and the corresponding products were then tested against isozymes hCA I, II and bCA IV. This series is characterized by a potent inhibitory activity on hCA II, most of the measured K_i_ values being in the low nanomolar range on this isoform, but in the micromolar range against hCA I and bCA IV. We can cite derivative **34a** as an example: the Barboiu group determined a K_i_ of 0.75 nM on hCA II while the values measured against hCA I and bCA were, respectively, 125 and 100 nM. There is indeed a strong selectivity for the human isoform II confirming the evidence that this scaffold could be a promising candidate for the development of selective hCA II inhibitors.

**Figure 16:**
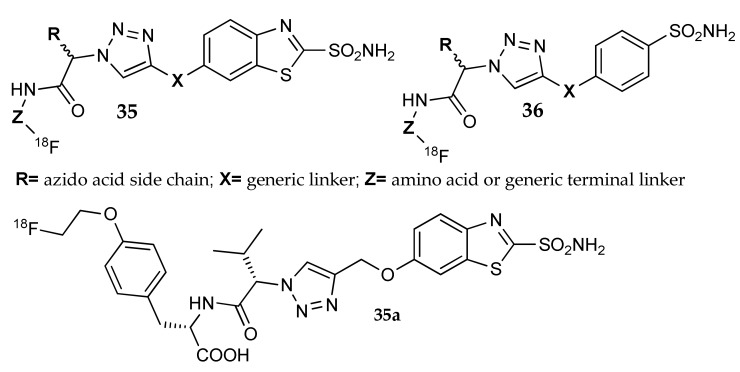
Sulfonamide derivatives carrying a triazole-amino acid as linker.

Kolb et al. patented a series of radioactively labelled sulfonamides [64], with high affinity for hCA IX, that could be potentially used as positron emission tomography (PET) tracers for imaging the hCA IX expression on the surface of cancer cells. The group identified a benzothiazole or *p*-substituted phenyl ring as ideal aromatic scaffolds to carry the sulfonamidic moiety and used click-chemistry to functionalize the inhibitor portion with different groups; a ^18^F atom was inserted on this tail as radionuclide. They designed four series of compounds and two of them (general formula **35** and **36**, Figure 16) were prepared using an amino acid derivative (azido acid) as building block for the construction of the triazole central ring. In addition, the use of a second amino acid as radionuclide-carrying portion was investigated. With such a strategy, these researchers were able to obtain a large library of compounds, with different chemical characteristics and many possible points of structural variation. Furthermore, the use of L or D amino and azido acids allowed the stereochemical control of the side chain and, consequently, its orientation toward the regions of the enzyme. One of the most promising compound, VM-4037A (**35a**), was tested in tumor xenograft models expressing hCA IX [65]. In addition, its biodistribution and radiation dose were determined in healthy human volunteers [66]; the high uptake of **35a** in liver and kidney indicated that the compound was not suitable for the detection of hCA IX in these tissues.

**Figure 17:**
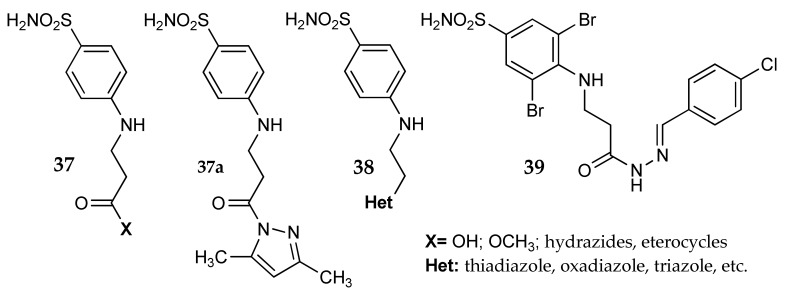
Structure of the β-alanine derivatives studied by the Matulis group.

A quite different approach was used by the Matulis group [67,68] to derive *p*-amino benzenesulfonamide. By reacting the *p*-amino group with acrylic acid, Rutkauskas et al. obtained a series of compounds where a β-alanyl moiety is directly linked to the aromatic scaffold (general formula **37** and **38**, Figure 17). In this instance, the amino acid linker was generated in situ and then functionalized on the free β-carboxy moiety. In addition, the versatile nature of this group allowed its transformation into heterocycles, such as triazoles, oxadiazoles or thiadiazoles [67]. Only in a few instances the activity of this first series of compounds (K_d_ values determined by a fluorescent thermal shift assay on CA I, II, VI, VII, XII and XIII) was below 1 μM. The functionalization of the alanyl nitrogen and substitution on the benzenesulfonamide ring were also investigated, adding other possible points of variation [68]. As one can imagine, this large number of different products showed a wide range of activities, with K_d_ values on CA I, II, XII and XIII from nanomolar to micromolar. The selectivity of some compounds was further tested on a larger panel of hCAs, with some interesting findings. For instance, **39** (Figure 17) showed good potency on CA VB and CA IX (K_d_ = 5 and 43 nM, respectively) while being almost inactive on the other isoforms. The crystallographic studies performed on **37a** and hCA II allowed the way the flexible β-alanyl linker was folded in the enzyme cavity (PDB code 4Q6E) to be understood.

**Figure 18:**
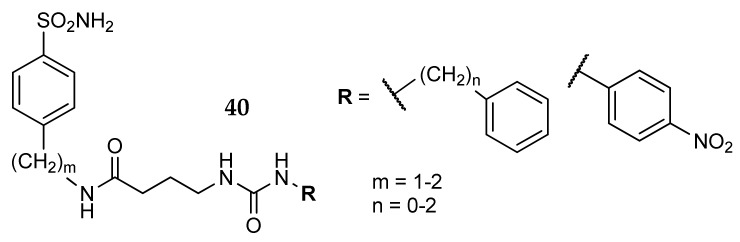
Benzenesulfonamide derivatives incorporating a GABA moiety.

Ceruso et al. [69] extended the chain using a γ-aminobutiric acid moiety as linker between two different benzenesulfonamides and various aromatic pendants. Compared to **37**, the amino acid orientation was inverted, leaving a free amino group that was reacted with aryl isocyanates. The molecules obtained (general formula **40**, Figure 18) were tested against 13 hCA isoforms, showing good inhibition potency against hCA I, II, VII and XII. This serie lacks isoform selectivity, but a deeper investigation on the effects of the chain length and of the chemical nature of the terminal aromatic pendants could potentially lead to more interesting derivatives.

**Figure 19:**
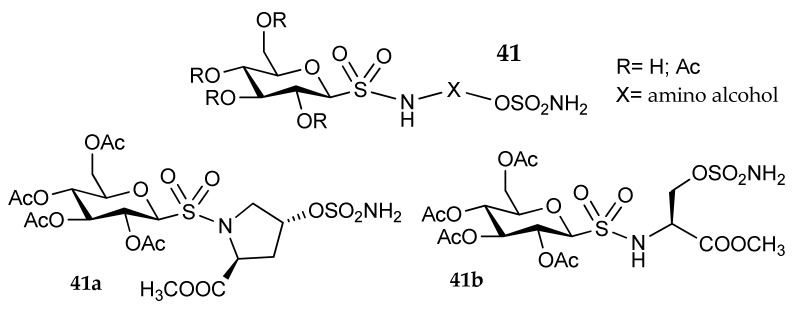
Sulfamate derivatives studied by Moeker et al. [70].

Moeker et al. [70] studied an innovative approach using a sulfamate as zinc binding group (ZBG). These researchers designed a series of glycosylated compounds with poor membrane permeability that could potentially lead to hCA IX selective inhibitors, owing to the extracellular localization of this enzyme. The general structure of these derivatives (**41**, Figure 19) is characterized by a free or acetylated glucose moiety, linked to a panel of primary and secondary amino alcohols through a sulfonamide bridge. Two hydroxylated amino acid (L-serine methyl ester and L-4-(*R*)-hydroxyproline methyl ester) were investigated as hydroxylated linkers and transformed into the corresponding sulfamate (**41a** and **41b**). In such a way, the ZBG is directly placed on the chiral amino acid side chain. All the synthesized molecules showed nanomolar K_1_ values against the tumor-associated isoforms hCA IX and XII [5,13], and lower potency against the off-target isozymes hCA I and II. Particularly interesting is the proline-derivative **41a**, with Ki values of 2 and 1 nanomolar respectively on hCA IX and XII and more than 350 times lower potency on hCA I and II. This innovative method is interesting not only from a synthetic point of view, but also because it allows the ZBG to be kept fixed and functionalizes both the amino and carboxy portions of the amino acid with different residues. 

These examples demonstrate that a chiral amino acid can serve either as building block either as linker between side pendants.

## 6. Dual Carbonic Anhydrase (CA) and Matrix Metalloproteinase (MMP) Inhibitors

Matrix metalloproteinases (MMPs) are zinc-containing endopeptidases, capable of degrading the extracellular matrix proteins; up-regulation of specific isoforms is associated with various pathologies, including some metastatic cancers [71,72,73]. These enzymes are potently inhibited by hydroxamates, but also other chemical groups are known to be effective Zn-chelating agents [74]. Since hCA IX and XII are overexpressed in tumors [5,13], the simultaneous inhibition of both enzymes should give a synergic effect. 

**Figure 20:**
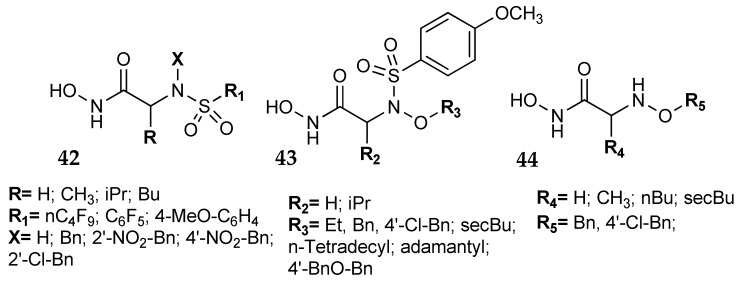
Dual Matrix Metalloproteinase–Carbonic Anhydrase (MMP–CA) inhibitors.

On these basis, dual inhibitors were designed [75,76], i.e., compounds able to interact with the binding regions of both metalloenzymes. Some amino acids were investigated as the structural backbone of the new dual inhibitors; the derivatization of the carboxy and amino moieties gave sulfonylated amino acid hydroxamates and N-alkyloxy amino acid hydroxamates (general formulas **42**–**44**, Figure 20). Compounds carrying both a hydroxamic and a protic-sulfonyl groups are, therefore, provided with two different chelating moieties that could potentially interact with both metalloenzymes. Compounds **42** were strong dual inhibitors with Ki values in the low-medium nanomolar range, their inhibitory properties against the two enzymes depending on some structural features [75]. For instance, compounds **42** carrying a NH moiety (**X** = H) were more active on CA (isoform I, II and IV) than on MMPs (isoforms 1, 2, 8 and 9). When X was a benzyl group, a potent activity was found on both enzymes, while a substituent on the benzyl moiety almost abolished activity on CAs. Unfortunately, these substances were not tested on the tumor-associated CA isoforms (IX and XII). 

Compounds **43** and **44** were in general less potent on both hCAs and MMPs, probably due to the complete absence of protic sulfonyl moiety that could behave as ZBG [76]. 

These studies confirm that amino acids, by showing good synthetic properties, are interesting synthons also for the design of hybrid inhibitors, where an important need is the possibility to easily modify many positions with different kind of substituents.

## 7. Other Approaches

**Figure 21:**
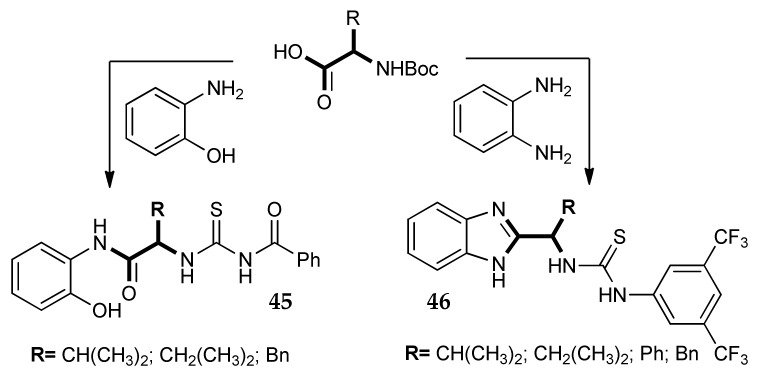
Thiourea CAIs built from amino acids.

Especially from a synthetic point of view, another interesting approach to the design of novel CAIs is the one reported in a paper by Korkmaz N. et al. [77]. These researchers, aiming to investigate innovative inhibitors, synthesized a series of thiourea derivatives lacking a classic ZBG. As we can note from general structures **45** and **46** in Figure 21, there are not pharmacophoric moieties that could be immediately associated with an inhibitory activity on the enzyme. Nevertheless, micromolar K_i_ values were measured for these series of compounds against hCA I and II. By analysing the structures, we can observe that lipophilic amino acids were used as linker between two aromatic portions. For **46**, the amino acid carboxy group reacted with *o*-phenylendiamine giving a benzimidazole ring. Therefore, compounds **46** can be considered as amino acid derivatives only from the synthetic point of view, since the carboxy group is lost with the formation of the heterocyclic ring. Compounds **45** and **46** were not extremely potent, but these synthetic pathways could be interesting for the side derivatization of sulfonamidic molecules.

**Figure 22:**
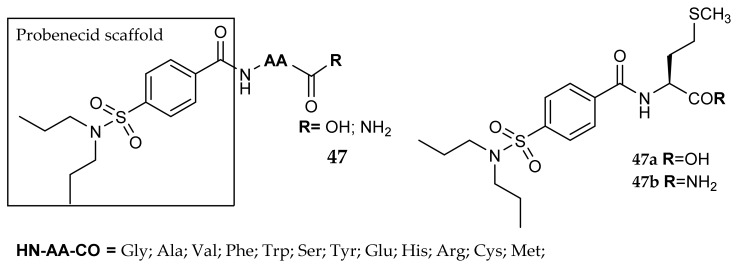
Probenecid amide derivatives.

Another scaffold lacking a classic chelating moiety is Probenecid, a drug used to treat gout and hyperuricemia. Probenecid, probably thanks to the free carboxy group that coordinates the zinc ion, possesses a weak inhibitory activity against CAs. Mollica et al. [78] investigated the effects of adding different L-amino acid, obtaining probenecid-based amide derivatives (general formula **47**, Figure 22). This modification shifted the carboxy group away from the aromatic ring and introduced a chiral linker. With the aim of studying the impact of the chemical modification of this terminal COOH, these researchers also synthesized the corresponding primary amides. All the compounds showed an interesting profile, their inhibitory activity against hCA IX and XII being higher than against the off-target isozymes hCA I and II. The transformation of the COOH group into CONH_2_ was also interesting, resulting in a remarkably lower affinity against hCA II. As an example, both the methionine derivatives **47a** and **47b** were very active against the two tumor-associated isoforms, but while the carboxy derivatives possess a low residual activity against hCA I and II, the corresponding amide is completely inactive toward these isoforms. This paper provides a useful overview of the application of the “amino acid tail approach” to an atypical zinc-chelating molecule, confirming the synthetic and pharmacological benefits that could be obtained from this method.

**Figure 23:**
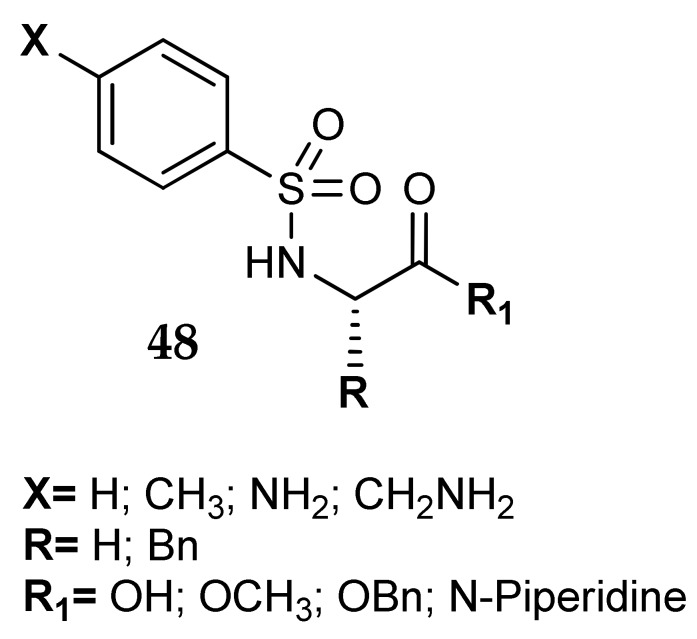
Glycine and phenylalanine N-sulfonamides studied by Fidan et al. [79].

In this section we report also the paper published by Fidan et al. [79], in which four aromatic sulfonamides were directly functionalized on the amidic NH_2_ group with glycine and phenylalanine (general formula **48**, Figure 23). The derivatization of the zinc binding group with substituents able to interact with the amino acids of the catalytic site was aimed at obtaining an enhancement in the activity of these secondary sulfonamides. However, it must be recalled that the architecture of the active site is very well conserved among the different CA isoforms, so this modification may not confer selectivity to such derivatives. In general, the new compounds did not show a better inhibition profile against hCA I and II as compared to their primary sulfonamide precursors. A substitution directly on the ZBG, probably due to steric hindrance, seems not to be a convenient way to enhance the potency of this kind of sulfonamides. Despite these negative results, this approach should be further investigated, maybe with longer linkers between the sulfonamidic nitrogen and the pendants. 

**Figure 24:**
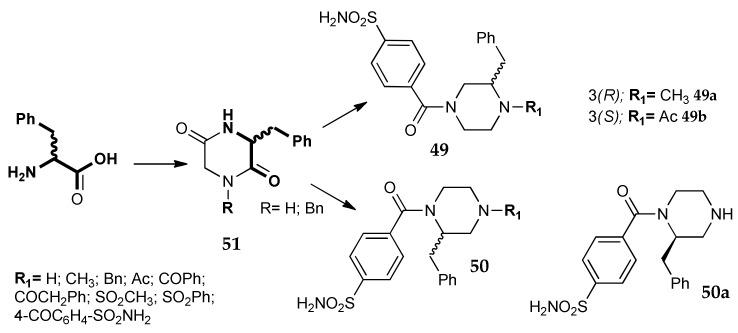
Piperazine derivatives studied by Chiaramonte et al. [23].

Chiral pool synthesis was used by Chiaramonte et al. in order to prepare enantiopure CA inhibitors [23]. Starting from l- or d-phenylalanine, both enantiomers of two series of 2-benzylpiperazines (**49** and **50**, Figure 24), carrying a sulfamoyl-benzoic moiety as ZBG, were obtained. The amino acid was directly used to build the piperazine scaffold, allowing the stereochemical control of the side benzyl group. With this method, it was possible to take advantage of the versatile synthetic properties of the synthon: the reduction of the intermediate diketopiperazine **51** converted the amino acid into an alkyl derivative without affecting the absolute configuration. The piperazines were further decorated on one nitrogen atom with the ZBG and with different alkyl/acyl/sulfonyl groups on the other one, with the aim to look for selectivity toward the CA isoforms. All the synthesized products were able to inhibit four pharmacological-relevant CA isozymes (hCA I, II, IV and IX), showing K_i_ values ranging from low nanomolar to micromolar; hCA IX was the least sensitive isoform. The hydrochloric salts of two basic piperazines (**49a** and **50a**, Figure 24), showing very good water solubility, were then selected for in-vivo tests in a rabbit model of glaucoma. Both derivatives were able to reduce IOP with a potency and efficacy similar to the reference drug dorzolamide, confirming that this series is promising for the development of new anti-glaucoma agents. The binding mode was further investigated with X-ray analysis and the structure of the complex of compound **49b**, bound in the hCA I active site, was solved (PDB code 6EVR). In the image, reported in Figure 1b (Section 1), it is possible to appreciate the interaction between the sulfonamidic group and the Zn^2+^, while the benzyl group is deeply inserted in the lipophilic cavity of the enzyme, where it establishes Van der Waals interactions with some hydrophobic amino acid residues. This synthetic method allowed the building of a chiral piperazine scaffold, incorporating the aminoacid-skeleton. Starting from different amino acids, it should be possible to modify the nature of this substituent and at the same time to control its stereochemistry.

## 8. Conclusions

Amino acids are useful synthons. Thanks to their characteristics, there are several potential applications in chemical synthesis. The possibility of using both proteinogenic and non-proteinogenic amino acids allow the preparation of many related derivatives, where the different side chains modulate the properties. The research of new CAIs or the structural modification of existing inhibitors is a dynamic field with many perspectives but also a lot of still unsolved challenges, the most important being the lack of selectivity of sulfonamides. The use of amino acids, or their derivatives, could sometimes provide a smart solution to both synthetic and pharmacological problems.

## Abbreviations

CA	carbonic anhydrase
hCA	human carbonic anhydrase
bCA	bovine carbonic anhydrase
CAIs	carbonic anhydrases inhibitors
MMPs	matrix metalloproteinases
IOP	intraocular pressure
Ala	alanine
Asn	asparagine
Asp	aspartic acid
Arg	arginine
β-Ala	beta-alanine
β-PhSer	beta-phenylserine
Boc	tert-butyloxycarbonyl
Cys	cysteine
DOPA	(3′,4′-dihydroxy)phenylalanine
Fmoc	fluorenylmethyloxycarbonyl
GABA	γ-aminobutyric acid
Gly	glycine
Gln	glutamine
Glu	glutamic acid
Ile	isoleucine
K_cat_	turnover number
K_d_	dissociation constant
K_i_	inhibition constant
Leu	leucine
Lys	lysine
Met	Methionine
PDB	protein data bank
PET	positron emission tomography
Phe	phenylalanine
PhGly	phenylglycine
Pro	proline
Ser	serine
Thr	threonine
Trp	tryptophan
Tyr	tyrosine
Val	valine
ZBG	zinc binding group
